# Genes showing altered expression in the medial preoptic area in the highly social maternal phenotype are related to autism and other disorders with social deficits

**DOI:** 10.1186/1471-2202-15-11

**Published:** 2014-01-14

**Authors:** Terri M Driessen, Brian E Eisinger, Changjiu Zhao, Sharon A Stevenson, Michael C Saul, Stephen C Gammie

**Affiliations:** 1 Department of Zoology, University of Wisconsin-Madison, Madison, WI, USA; 2 Neuroscience Training Program, University of Wisconsin-Madison, Madison, WI, USA

**Keywords:** Autism, Bipolar disorder, Depression, Schizophrenia, CNS development, Postpartum females, Medial preoptic area, Modular Single-Set Enrichment Test (MSET)

## Abstract

**Background:**

The mother-child relationship is the most fundamental social bond in mammals, and previous studies indicate that the medial preoptic area (MPOA) contributes to this increase in sociability. It is possible that the same genes that lead to elevated sociability in one condition (the maternal state) might also be dysregulated in some disorders with social deficits (e.g. autism). In this study, we examined whether there was enrichment (greater than chance overlap) for social deficit disorder related genes in MPOA microarray results between virgin and postpartum female mice. We utilized microarrays to assess large scale gene expression changes in the MPOA of virgin and postpartum mice. The Modular Single Set Enrichment Test (MSET) was used to determine if mental health disorder related genes were enriched in significant microarray results. Additional resources, such as ToppCluster, NIH DAVID, and weighted co-expression network analysis (WGCNA) were used to analyze enrichment for specific gene clusters or indirect relationships between significant genes of interest. Finally, a subset of microarray results was validated using quantitative PCR.

**Results:**

Significant postpartum MPOA microarray results were enriched for multiple disorders that include social deficits, including autism, bipolar disorder, depression, and schizophrenia. Together, 98 autism-related genes were identified from the significant microarray results. Further, ToppCluser and NIH DAVID identified a large number of postpartum genes related to ion channel activity and CNS development, and also suggested a role for microRNAs in regulating maternal gene expression. WGCNA identified a module of genes associated with the postpartum phenotype, and identified indirect links between transcription factors and other genes of interest.

**Conclusion:**

The transition to the maternal state involves great CNS plasticity and increased sociability. We identified multiple novel genes that overlap between the postpartum MPOA (high sociability) and mental health disorders with low sociability. Thus, the activity or interactions of the same genes may be altering social behaviors in different directions in different conditions. Maternity also involves elevated risks for disorders, including depression, psychosis, and BPD, so identification of maternal genes common to these disorders may provide insights into the elevated vulnerability of the maternal brain.

## Background

In many species, maternal care is critically important for sustaining offspring until they are self-sufficient, and the extent of nurturing behavior contributes to the physiological and behavioral development of the offspring [1]. The social bond formed between a mother and offspring is the most fundamental social bond created in mammals, and offspring are innately rewarding to mothers [2]. In rodents, maternal care is facilitated in part by changes in circulating hormones during late pregnancy, as well as the tactile and olfactory sensory input from the offspring [3]. Large scale gene expression changes occur in the CNS that support the emergence of the maternal phenotype and recent work has used microarray approaches to document this plasticity [4,5].

While many brain regions support nurturing behaviors, the medial preoptic area (MPOA) plays a central role in both the onset and the maintenance of maternal care during the early postpartum period [3,6,7]. Lesions to the MPOA, as well as temporary inactivation of the MPOA using GABA agonists and bupivacaine inhibits components of maternal behaviors during the early postpartum period [8-10]. Lesions or temporary inactivation of the MPOA also affect socially motivated behaviors. The rewarding aspect of pup exposure is dampened following either lesions or bupivacaine injections in the MPOA [11,12], as are sexually motivated social behaviors [13,14]. The MPOA is interconnected with the septum and amygdala, and receives afferent input from the bed nucleus of the stria terminalis, paraventricular nucleus (PVN), and medial prefrontal cortex [15,16]. In addition, MPOA cells activated during maternal care project to the anterior hypothalamic nucleus, ventral tegmental area, periaqueductal gray, and retrorubral field [17].

Although the MPOA plays a key role in maternal behavior, studies examining broad changes in gene expression are needed to more fully understand this critical maternal brain region. In the present study, we used high density oligonucleotide microarrays to examine large scale gene expression changes in the MPOA of postpartum and virgin females. An initial analysis of results indicated that a substantial amount of mental health disorder related genes appeared in some of the most significant microarray data. We therefore utilized a new tool, the Modular Single-Set Enrichment Test (MSET), to directly assess if genes associated with mental health disorders were enriched in our results by appearing at a rate higher than expected due to random chance [18]. While there are many enrichment tools publicly available [19], MSET was employed because it allows the user to compare microarray results with independently curated disease associated gene lists that are not commonly associated with other enrichment tools. Though the Broad Institute’s GSEA software tool does allow the user to input gene lists of interest, there are some key differences [20]. While GSEA uses the entire dataset to assess enrichment for genes of interest, MSET allows the user to input a definite set of genes with a specific p-value cutoff, thereby allowing the analysis of only the most significant genes. Further, the computations necessary to assess enrichment using GSEA can be complex and unclear for the average user, whereas MSET employs a simple randomization test [18].

The first study to use MSET identified enrichment for autism spectrum disorder (ASD) related genes, as well as genes associated with other mental health disorders, in a microarray conducted in the lateral septum (LS) between postpartum and virgin females [18]. Social impairment is one of the characteristic symptoms of ASD [21,22], and it is possible that genes associated with social impairment are altered in the postpartum brain to promote social bonding with offspring. We tested this in the MPOA by assessing microarray results for enrichment of specific mental health disorders, such as ASD, bipolar disorder, depression, and schizophrenia, and confirmed some of those gene expression changes using qPCR. Two additional analytical tools, the NIH’s DAVID and ToppCluster, were utilized to determine if specific biological pathways were overrepresented in significant microarray results [23,24]. Gene co-expression was also assessed using the weighted gene co-expression network analysis (WGCNA) to identify potential connectivity between microarray genes [25,26].

## Results

### Genes differentially expressed between virgin and lactating females

Using Probe Logarithmic Intensity Error analysis (PLIER), we identified 734 annotated genes with a nominal p-value < 0.01. A full list of all 35,557 targets, p-values, and the relative expression for each gene are available in Additional file 1. The raw and summarized expression data for this publication have been deposited in NCBI’s Gene Expression Omnibus [27].

### Enrichment for mental health disorder related genes using MSET analysis

**Figure 1:**
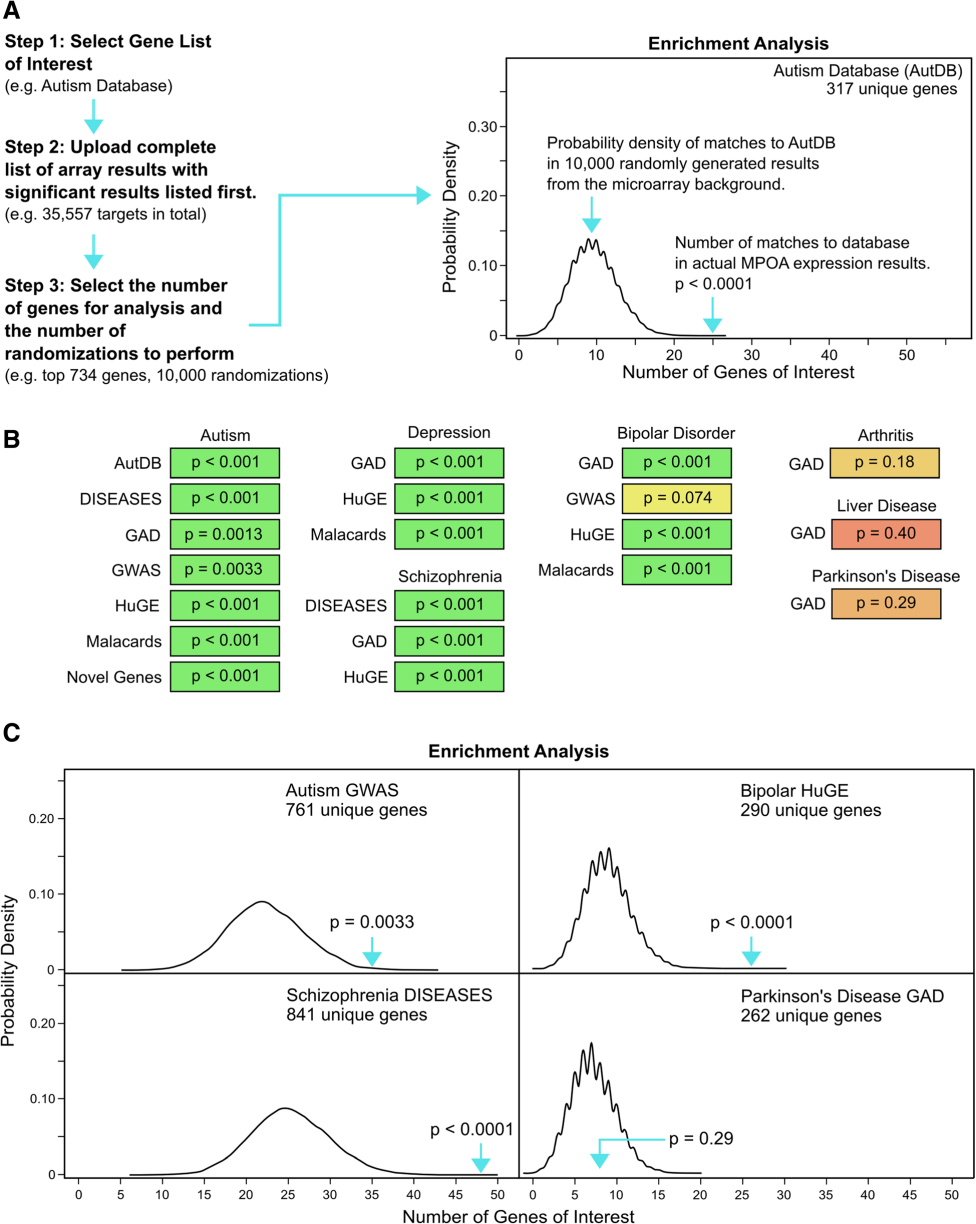
**Overview of MSET procedure, and enrichment values for mental health disorder realted gene lists. (A)** Overview of the MSET procedure with examples of gene lists selected for our analysis. After entering the desired number of randomizations to perform, a probability density distribution of matches to the gene list of interest is generated. The second blue arrow shows how many matches to the gene list of interest were found in our significant microarray results and the corresponding enrichment value. The y-axis represents the probability of matches between gene lists of interest and 10,000 randomly generated lists, and the x-axis denotes the number of genes of interest found in each randomly produced list. **(B)** Enrichment values for all gene lists tested relating to ASD, BPD, depression, and schizophrenia, and the control lists from GAD associated with arthritis, liver disease, and Parkinson’s disease. P-values were shaded based on their degree of significance, with green being the most significant and red being the least significant. MSET analysis detected overall enrichment for ASD, depression, and schizophrenia related genes in the top 734 genes from the microarray. Three of the four BPD associated gene lists revealed significant enrichment within the microarray results, but only a trend toward significance was found using the GWAS list. The control lists were non-significant, providing evidence for the specificity of MSET analysis. **(C)** Examples of the probability density distribution for four gene lists relating to ASD, BPD, schizophrenia, and Parkinson’s Disease. Arrows mark the number of genes of interest found in the top 734 genes from the microarray data.

Gene lists relating to ASD, bipolar disorder (BPD), depression, and schizophrenia were compiled from five different online databases: the Autism Database AutDB [28], the Copenhagen DISEASES database [29], the Genetic Association Database (GAD) [30,31] the HuGE Navigator [32] and the Human Malady Compendium (MalaCards) [33]. Two additional ASD related gene lists were assembled from recent genome wide association studies: Autism GWAS [34], and Autism Novel Genes [35]. To ensure that no two gene lists associated with a specific disorder were nearly identical, pairwise comparisons were conducted to determine the degree of similarity (data not shown). In addition, any result with a skewed density plot was not included in the final analysis (data not shown). Enrichment for mental health related genes was assessed in the top 734 significant genes from the microarray experiment. An overview of the MSET procedure and interpretation of the resulting probability density distributions can be found in Figure 1A.

In total, 7 independent ASD gene lists were assembled and tested. MSET analysis found significant enrichment for all 7 lists (p < 0.05) in the top microarray results, indicating that ASD related genes appear in significant microarray data at a rate higher than what would be due to random chance (Figure 1B). Combined, 98 ASD related genes were found in the significant microarray data (Additional file 2), with 34 of those 98 ASD related genes appearing in at least two of the assembled gene lists (Table 1). The appearance of genes on more than one list indicates there may be more evidence to support an association with ASD.

**Table 1:** ASD, BPD, depression, and schizophrenia related genes (p-value <0.01) found in two or more independent lists

ASD related genes						
Acsl4	Apbb2	Atp2b2	Atp10a	Bcl2	Cacna1g	Cdh13
Cntnap2	Csn1s1	Dock3	Gabra2	Grik1	Grik3	Hcrtr2
Igf1	Mfsd6	Nos1	Nrp2	Nxph1	Oprl1	Oxtr
Park2	Pcdh9	Prkcb	Ptchd1	Rims3	Rora	Rps6ka6
Shank3	Spon1	Tsc1	Vgf	Vldlr	Wnk3-ps	
BPD related genes						
Ace	Alg9	Bcl2	Chrna3	Chrnb2	Cntnap2	Cry1
Cry2	Dbp	Dscam	Dusp6	Fbn1	Fkbp5	Gabra2
Gabre	Gabrq	Grik1	Igf1	Ncan	Nos1	Nr1d1
Nr4a3	Penk	Per3	Rfx4	Rora	Slc1a4	Zdhhc8
Depression related genes						
Ace	Acs14	Adcyap1r1	Alox5ap	Cdh13	Creb1	Cry1
Cry2	Dbp	Fkbp5	Grik1	Grik3	Hspa1a	Htt
Nos1	Nr1d1	Oxtr	Per3	Rora	Smpd1	Stat3
Schizophrenia related genes						
Ace	Bcl9	Cdc42se2	Chi3l1	Chrna3	Chrnb2	Cntnap2
Creb1	Egr1	Fxyd6	Gabra2	Glul	Grik1	Grik3
Hspa1a	Itih3	Ncan	Nos1	Notch3	Olig2	Oxtr
Per3	Pla2g4c	Rgs10	Shank3	Slc1a4	Slc6a9	Tacr3
Ucp2	Vldlr	Zdhhc8				

Depression linked gene lists were assembled from four databases, but one gene list (DISEASES) was too small to yield a probability density plot that was not skewed (data not shown). It should also be noted that the Genetic Association Database (GAD) depression list contains genes related to “depression” and “major depression”. Significant enrichment for depression related genes was found in the MPOA results using all three depression related gene lists (Figure 1B). A total of 40 depression related genes were found in the significant microarray genes (Additional file 2), with 21 of those 40 depression related genes appearing in at least two of the three gene lists assembled (Table 1).

Significant enrichment for BPD-related genes was detected using three of the gene lists tested, and reached near significance with the assembled GWAS gene list (p = 0.074) (Figure 1B). Combined, 51 BPD related genes were found in the significant microarray genes (Additional file 2), with 28 BPD related genes appearing in at least two of the four gene lists assembled (Table 1).

Schizophrenia related genes were also highly enriched in the top microarray results (Figure 1B). A total of 74 schizophrenia related genes were found in the microarray results (Additional file 2), with 31 of those 74 genes appearing in at least two of the three lists (Table 1).

To assess the specificity of MSET within the MPOA, gene lists were assembled from the GAD online database for Parkinson’s disease, arthritis, and liver disease. Enrichment for these diseases was not detected in the significant microarray data (p > 0.05), which suggests that the postpartum brain is only linked to a subset of disorders (Figure 1B). Examples of the probability density distributions for significantly enriched data sets and a non-significant data set can be found in Figure 1B.

### QPCR verification of microarray results

Real-time qPCR confirmation of significant microarray results were conducted using a subset of genes identified in MSET analysis results, as well as genes identified in multiple microarrays comparing lactating and virgin females. The 13 genes tested using qPCR, as well as their abbreviation, p-value, and fold change from the microarray, can be found below. Fold changes higher than 1 indicate that expression is increased in postpartum females relative to virgin females, and fold changes less than 1 indicate that expression is decreased in postpartum compared to virgin females. A complete list of microarray data, including individual gene p-values and fold changes can be found in Additional file 1. Angiotensin I converting enzyme (Ace; p = 0.019; fold change = 0.91), B cell leukemia/lymphoma 2 (Bcl2; p = 0.00055; fold change = 1.08), contactin associated protein-like 2 (Cntnap2; p = 0.000015; fold change = 0.85), gamma-aminobutyric acid (GABA) A receptor, subunit delta (Gabrd; p = 0.013; fold change = 1.08), gamma-aminobutyric acid (GABA) A receptor, subunit epsilon (Gabre; p = 0.00046; fold change = 0.73), gamma-aminobutyric acid (GABA) A receptor, subunit theta (Gabrq; p = 0.0021; fold change = 0.84), glutamate-ammonia ligase (Glul; p = 0.0029; fold change = 1.12), nitric oxide synthase I (Nos1; p = 0.0021, fold change = 0.85), oxytocin receptor (Oxtr; p = 0.0059, fold change = 1.14), reelin (Reln; p = 0.014; fold change = 0.885), SH3/ankyrin domain gene 3 (Shank3; p = 0.00011; fold change = 1.09), suppressor of cytokine signaling (Socs2; p = 0.0000021, fold change = 1.63), and tuberous sclerosis 1 (Tsc1; p = 0.00068, fold change = 0.92).

**Figure 2:**
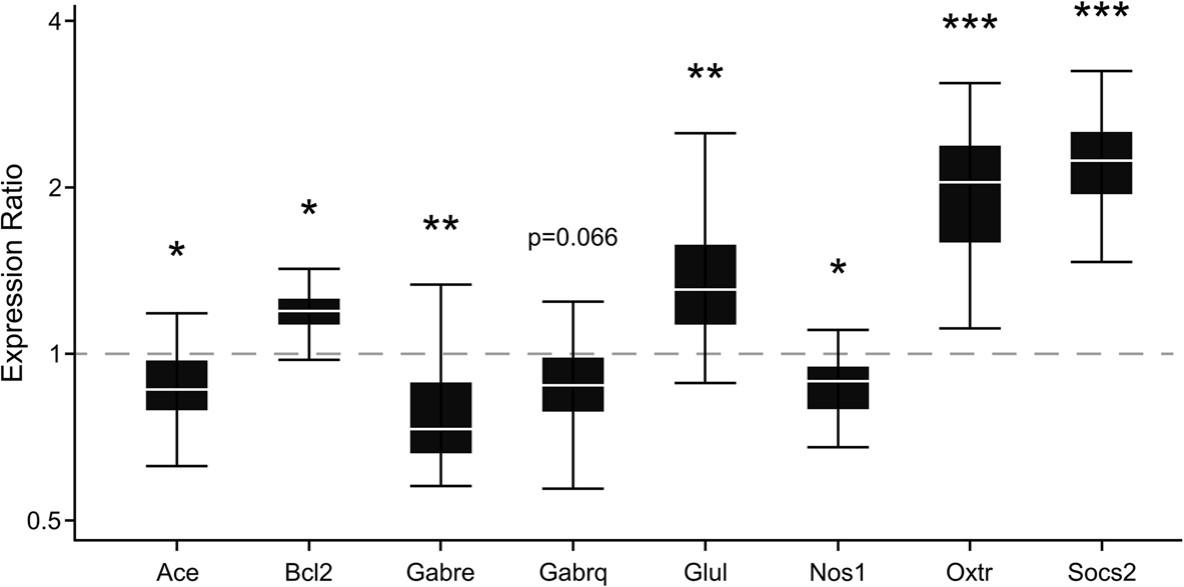
**Significant and near-significant qPCR results for a subset of genes differentially expressed in the MPOA.** The relative expression (y-axis) represents a ratio of gene expression in postpartum versus virgin females, using Hmbs and Hprt as reference genes. Ratios over one indicate genes with higher expression values in postpartum females, while ratios less than one indicate genes with lower expression values in postpartum females. The box whisker plots demarcate the range (whiskers), interquartile range (box), and median (solid white line) for each gene tested. In confirmation of microarray results, Ace, Gabre, and Nos1 were significantly down-regulated in lactating females, and Bcl2, Glul, Oxtr, and Socs were significantly up-regulated in lactating females. While Gabrq was significantly down-regulated in microarray results, there was a trend toward significance in qPCR results. *p < 0.05; **p < 0.01; ***p < 0.001.

Consistent with the findings from the microarray, qPCR results indicated that Ace (p = 0.027), Gabre (p = 0.008), and Nos1 (p = 0.014) were significantly down-regulated in the postpartum group compared to virgins, while Gabrq trended toward significant down-regulation (p = 0.066) (Figure 2). Bcl2 (p = 0.001), Glul (p = 0.006), Oxtr (p < 0.001), and Socs2 (p < 0.001) were significantly up-regulated in lactating females, which was consistent with the microarray findings (Figure 2). Although the p-value did not reach significance using qPCR, the direction of change for Cntnap2, Gabrd, Reln, Shank3, and Tsc1 was identical to that found in the microarray, suggesting some consistent, but possibly more subtle, alterations are occurring in the postpartum brain for these genes.

### CNS development and Ion channel activity are enriched in microarray results

**Figure 3:**
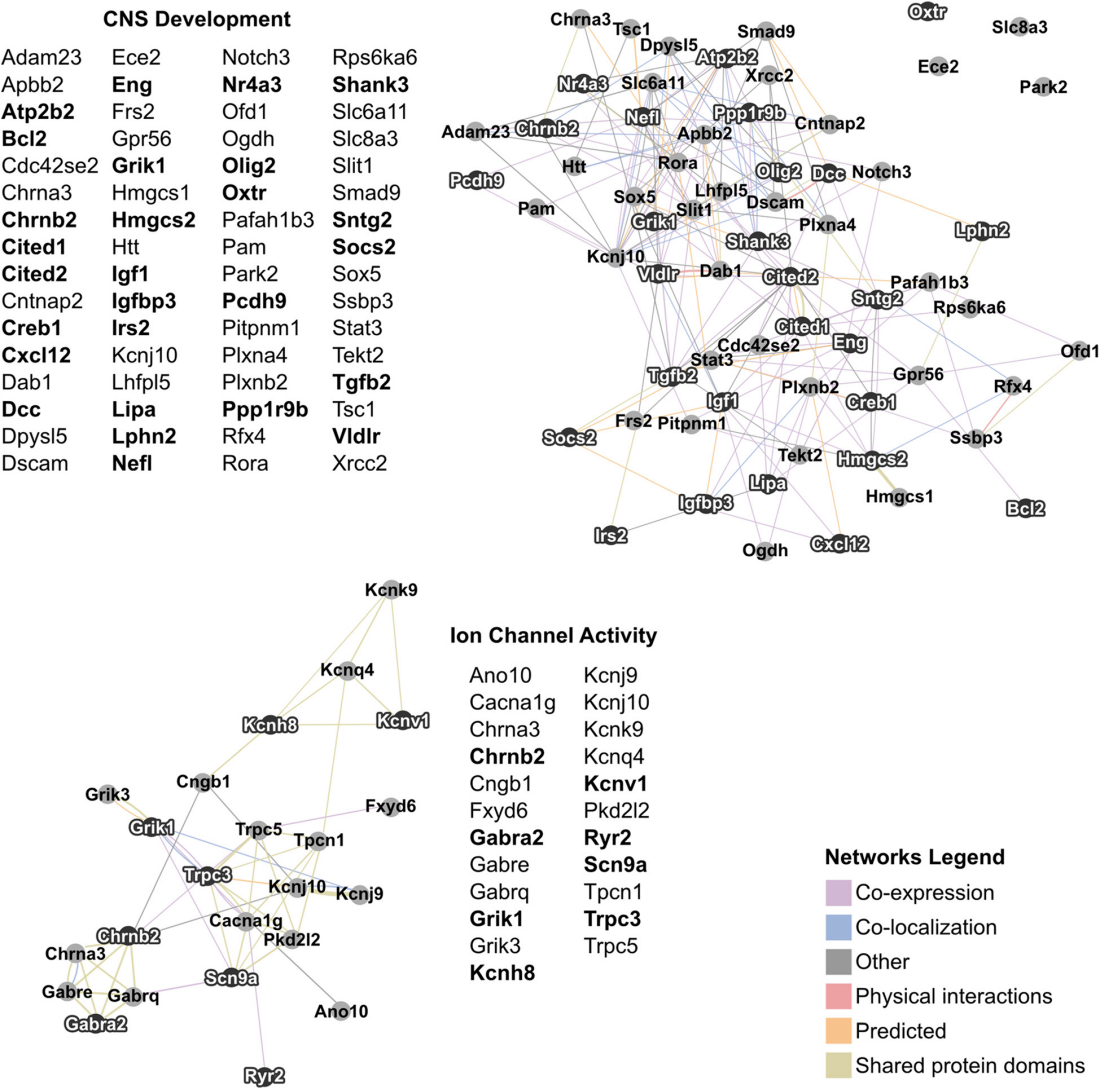
**Networks of genes related to CNS development and ion channel activity in significant MPOA results.** CNS development and ion channel activity related genes identified by ToppCluster and NIH DAVID were exported into GeneMania for visualization. A list of genes found within each individual network are found to the side of the cluster. Bolded terms in the list represent genes that are up-regulated in postpartum compared to virgin females, and non-bolded terms correspond to genes down-regulated in postpartum versus virgin females. Within the cluster itself, up-regulated genes have white text with black nodes, and down-regulated genes have black text with gray nodes. The distance between two nodes corresponds with the strength of their interactions, with the type of interaction denoted by the color found in the network legend at the bottom right of the figure.

The software tools ToppCluster and NIH DAVID were used to evaluate enrichment for specific gene clusters within the top 734 most significant microarray results. The most enriched cluster from the ToppCluster results was related to CNS development. Using NIH DAVID, multiple small clusters relating to neuronal differentiation, regulation of neuron projection, CNS development, and cellular morphogenesis were found, though none of these individual clusters were significantly enriched. These genes were pooled with ToppCluster results and the connectivity network can be found in Figure 3. NIH DAVID’s functional annotation tool also identified enrichment for ion channel activity within the significant microarray results (Figure 3). The distinct clusters found by ToppCluster and NIH DAVID are most likely due to different statistical methods and gene lists used by each database.

### MicroRNA and transcription factor binding sites in the MPOA of postpartum versus virgin females

Using significant microarray genes with a p < 0.01, ToppCluster identified 7 different miRNA binding sites, with 9 different miRNAs listed: MIR-32, MIR-92, MIR-96, MIR-129-5p, MIR-140-3p, MIR-141, MIR-200A, MIR-218, and MIR-1271. For two binding sites, two miRNAs were listed. For example, the conserved binding site UUGGCAC can be bound by MIR-96 and MIR-1271. When genes with a p < 0.05 were entered into ToppCluster, a total of 78 miRNAs were identified (Additional file 3). Interestingly, ToppCluster did not identify enrichment for any transcription factor binding sites in the most significant microarray genes, but utilizing the Animal Transcription Factor Database, we identified 37 transcription factors, 17 co-factors, and 7 chromatin remodeling factors differentially regulated within our results, which were then visualized using WGCNA.

### Co-regulated network of genes identified using WGCNA

**Figure 4:**
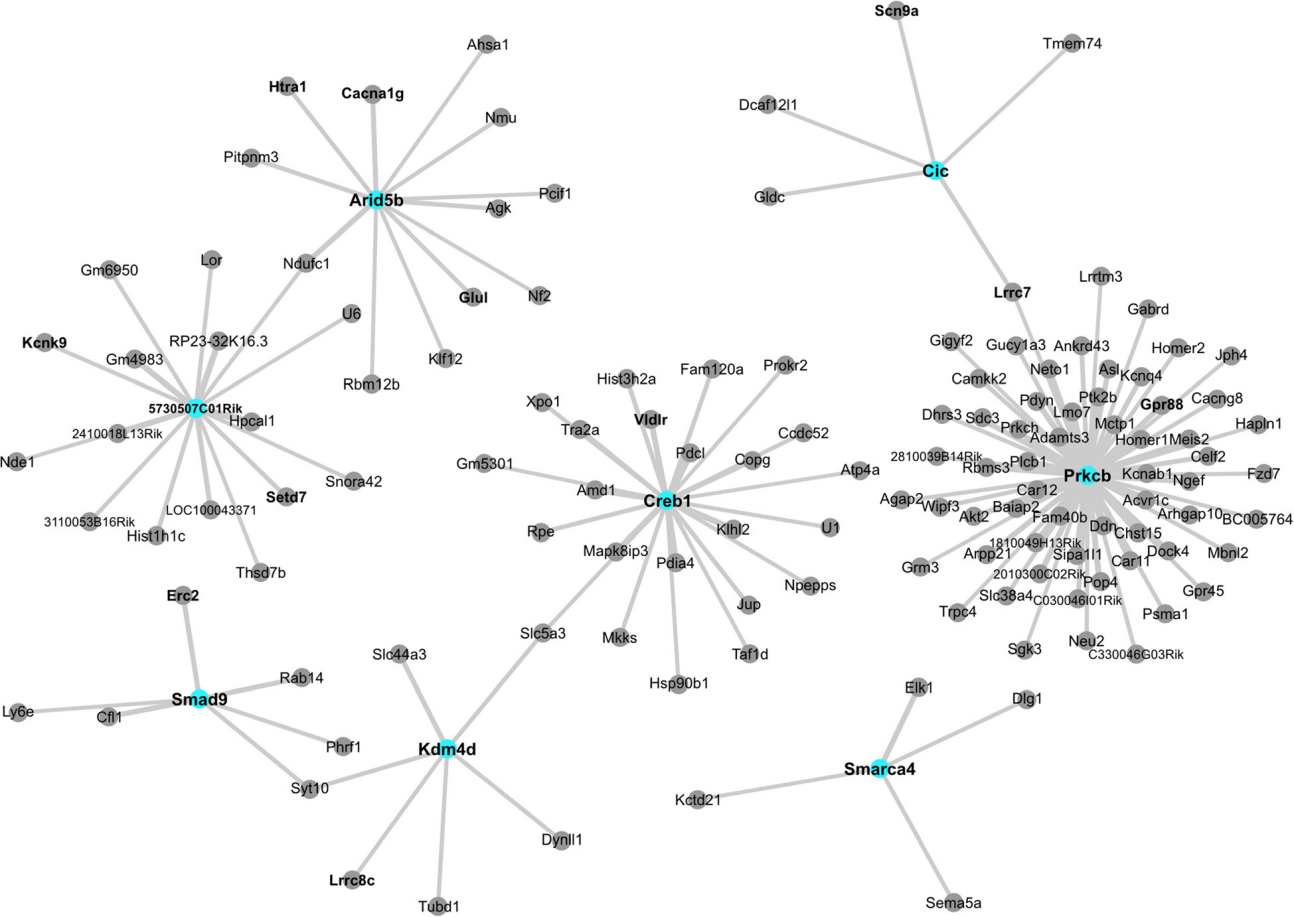
**WGCNA generated correlations containing transcription factors found in the module correlated with the postpartum phenotype.** Of the two modules generated using WGCNA, one was highly correlated with the postpartum phenotype. All gene-to-gene correlations involving transcription factors were selected from the module associated with the postpartum phenotype, and the network was visualized using Cytoscape. Edge width and size are proportional to the weight of the correlation between two genes. Blue nodes with bolded text are highly significant (nominal p < 0.01) transcription factors, co-factors, and chromatin remodeling factors correlated to a subset of other variable genes (nominal p < 0.05) from the microarray. Bolded text with gray nodes are genes found in other enriched gene lists, and include genes linked to mental health disorders, CNS development, and ion channel activity. Some transcription factors were also found in gene lists linked to mental health disorders, CNS development, and ion channel activity, but have been marked as transcription factors for this visualization. A full list of nodes and the weighted interactions for these genes can be found in Additional file 4.

Using gene expression levels previously calculated in this study, we used WGNCA to examine networks of genes that were co-regulated in relation to the reproductive state of the females. Analysis revealed two individual modules (blue and turquoise), with the blue module highly correlated to the postpartum phenotype (R^2^ = 0.85, student p-value = 0.0005), and the turquoise module highly correlated to the virgin phenotype (R^2^ = 0.95, student p-value = 0.000003). Of the 61 transcription factors highly significant in the microarray results, 54 were found across the two individual modules with 8 found in the module highly correlated to the postpartum phenotype. All gene-to-gene correlations involving transcription factors were selected from the module associated with the postpartum phenotype, and the network was visualized using Cytoscape (Figure 4).

A portion of genes relating to mental health disorders and CNS development already described in this paper were found to be co-regulated with many of these transcription factors. Specifically, 30 out of the 65 genes found relating to CNS development, and 75 genes relating to mental health disorders were correlated with transcription factors in both WGCNA modules (Additional file 4). Of the 61 transcription factors, co-factors, and chromatin remodeling factors significantly altered in postpartum females, only 6 were not found to be in any of the resulting modules: Chd5, Nr4a3, Supt3h, Wdr77, Zfhx4, and Zfp592. Hdac5 was located within one of the modules, however, it was removed from analysis due to inconsistent expression changes in microarray results.

## Discussion

This study used Affymetrix microarrays and qPCR to evaluate endogenous changes in gene expression in the MPOA of virgin versus postpartum female mice. A significant amount of genes associated with ASD, BPD, depression, and schizophrenia were found in top microarray results using a new enrichment analysis tool, MSET. These results compliment a recent study that found enrichment for genes linked to ASD in the postpartum versus virgin LS [18]. These select mental health disorders are all associated with some aspect of social impairment, suggesting that genes found in both the MPOA microarray and mental health disorder gene lists may be associated with sociability. Specifically, we suggest that these genes may be altered in maternal females to promote maternal bonding and sociability, while they may be dysregulated in individuals with a social deficit. Microarray results also indicated that genes associated with neural development and ion channel activity were enriched in the postpartum CNS, as were a large subset of transcription factors.

### Significant microarray results are enriched for genes associated with psychological disorders

Significant enrichment for ASD related genes was found in the most significant microarray results (734 genes) for all ASD associated gene lists tested. The high degree of enrichment for ASD linked genes may reflect the social deficits of some individuals with ASD and the role of the MPOA in social behaviors. Decreased affiliative behaviors, including deficits in the formation of a strong social bond and motivation to interact in social situations, is a characteristic commonly found in individuals with autism [21,22]. The social reciprocity necessary for the formation of parent-child bonds can fail to develop in some autistic patients [36]. In rodents, lesions to the MPOA in postpartum females inhibit the rewarding aspect of pup exposure as well as maternal behaviors in general [7-12], and inhibit sexual social behaviors in both male and female rats [13,14]. Collectively, this indicates that the MPOA contributes to socially motivated behaviors, and those social behaviors may in part be regulated by genes that are altered in individuals with ASD.

All depression linked gene databases, and three of the four BPD related lists analyzed with MSET found significant enrichment for genes related to these mental health disorders in the top microarray results. The occurrence of depressive symptoms is relatively common during the postpartum period (1-10% of women) [37], and can be detrimental to the child and mother. Studies have indicated that depressed mothers have a lower degree of positive social interactions (such as reading or telling stories) with their children, which can affect aspects of child development later on in life [38]. Individuals with BPD experience varying degrees of manic and depressive episodes and may have altered social functioning compared to control individuals [39]. In addition, there is an increased risk for postpartum women to develop BPD [40]. Individuals that are affected with BPD before parturition have a much higher risk of developing postpartum psychosis than others [41]. Postpartum psychosis is found in approximately 1 out of every 1000 new mothers and is characterized by paranoid delusions, mood swings, and confused thinking. Investigating altered expression for a broad range of genes in the postpartum brain may provide insights into understanding these diseases in the postpartum female.

Significant enrichment was found for all schizophrenia related gene lists in our microarray results. Research has indicated that social cognition is commonly impaired in individuals with schizophrenia, which includes the inability to comprehend emotional facial expressions, difficulty in maintaining eye contact, and decreased empathy [42-44]. Deficits in social cognition can greatly inhibit social interactions and socially motivated behaviors. One possibility is that the social aspect of schizophrenia is the main force behind the overall enrichment for schizophrenia related genes in the MPOA of postpartum versus virgin females. Oxytocin signaling has been implicated in many social behaviors [45-47], and has recently been applied to autism and schizophrenia research. Interestingly, intranasal administration of oxytocin to individuals with schizophrenia improves social perception and the ability to correctly recognize emotions [48-50]. In addition, social functioning was partially restored in animal models of schizophrenia following oxytocin administration [51].

The linkage of genes to mental health disorders often occurs via single nucleotide polymorphism studies in humans, but some studies have been able to find changes in gene expression or protein expression between individuals with mental health disorders and controls. Individuals with autism and bipolar have been found to have decreased levels of Bcl2 protein in the frontal cortex compared to controls [52-54]. Glul enzymatic activity was decreased in individuals with schizophrenia compared to controls in the cortex [55]. Also, alterations in Nos1 expression has been found in different brain regions of schizophrenics compared to controls [56]. All three of these genes showed altered expression in the postpartum brain and future studies evaluating how altered expression is linked to differences in behavior will be valuable.

A recent microarray conducted in the LS of virgin and postpartum females also found enrichment for ASD, BPD, depression, and schizophrenia related genes in the most significant microarray results [18]. Though a meta-analysis using the LS and MPOA microarray is underway, we can already see that there is a minor degree of overlap between ASD associated genes in both microarrays. Specifically, 98 unique ASD related genes were found in the significant MPOA microarray, while 160 ASD related genes were found in significant LS microarray data. The overlap between those two lists is relatively small (11 genes in total), which may be due to the different connectivity and functionality of the MPOA and LS in postpartum females. It is intriguing that such a high degree of mental health disorder related genes are altered during the transition to a postpartum state in two different brain regions associated with maternal care, and future studies investigating the role of these genes in regards to sociability are warranted.

### Genes related to CNS development are altered in the postpartum MPOA

Enrichment analysis using the software tools ToppCluster and NIH DAVID identified a large subset of genes linked to CNS development that were differentially regulated in the MPOA of postpartum females. These results contribute to a growing body of evidence indicating that alterations in CNS development occur during the postpartum period [57]. Specifically, administration of a hormone regimen similar to late pregnancy led to a decrease in cell proliferation in the hippocampus of “postpartum” females [58]. Decreased cell proliferation, which can be a marker for cell differentiation, was also found to be linked to pup exposure and lactation [59]. In addition, a recent microarray study in the LS suggests that cells may not fully differentiate until the postpartum period [5].

We confirmed a subset of genes linked to CNS development and mental health disorders using qPCR. Microarray analysis indicated that Bcl2 was up-regulated in lactating females compared to virgins, and this was confirmed using qPCR (p = 0.001). Bcl2 is an integral membrane protein found in the mitochondria, endoplasmic reticulum, and plasma membrane [60]. The anti-apoptotic actions of Bcl2 have been linked to brain development, and it is expressed at elevated levels concurrent with a period of neuronal remodeling in the embryonic murine CNS [61]. Though the expression of Bcl2 is decreased in the nervous system following birth, the persistent expression of Bcl2 indicates that it may continue to maintain the nervous system during adulthood [62,63]. For example, following brain injury, over-expression of Bcl2 has been found to increase activation of neural progenitor cells and reduce apoptosis of newly formed neurons [64].

Expression of Oxtr mRNA was significantly up-regulated in the postpartum MPOA compared to virgin females (p < 0.001). Oxtr is transiently expressed in some brain regions of developing rodents, indicating Oxtr potential involvement in developmental processes [65,66]. Oxtr has been previously found in glial tumors [67] and may induce cell proliferation via MAPK pathways [68]. The up-regulation of Oxtr in the postpartum MPOA is also consistent with previous studies investigating Oxtr and lactation. Oxtr knockout mice exhibit deficits in maternal behaviors [69], and antagonizing Oxtr delays the onset of maternal care [70]. Oxytocin signaling is also associated with social affiliation and social recognition. Injection of oxytocin into the brain of adult rats increases social contact time [71], and oxytocin injections in the MPOA of male rats prolong the period of social recognition [72].

Suppressor of cytokine signaling 2 (Socs2) is the most abundant member of a group of proteins that negatively regulate cytokine signaling in the CNS [73]. Socs2 is expressed at high levels during neurogenesis [74], and the level of Socs2 expression in neural progenitor cells can dictate the ratio of astrocytes and neurons produced during differentiation [75]. Socs2 expression was found to be significantly up-regulated in the LS of postpartum females compared to virgin and postpartum females deprived of pups, indicating that sensory input from offspring is a key regulator of Socs2 expression [5]. Our finding that Socs2 is up-regulated in the MPOA of postpartum females compared to virgins complements this finding. Given the up-regulation of Socs2 in two different brain regions during the postpartum period, future studies examining Socs2 expression in other maternally linked brain regions would provide a more complete functional profile of this gene during the postpartum period.

Nos1 was not found in ToppCluster or NIH DAVID gene lists linked to development, but has been previously associated with CNS development. Expression of Nos1, a neuronal nitric oxide synthase, was decreased in lactating compared to virgin females (p =0.027). The synthesis of nitric oxide, a neuromodulator, from L-arginine is dependent on activation of nitric oxide synthase by Ca2+/calmodulin [76]. Nos1 staining is prominent between embryonic days 15-19 in the murine CNS, and declines following parturition [77], with the highest Nos1 expression coinciding with developmental synaptogenesis [78]. Stimulation of Nos1 enhances neurite outgrowth, and over-expression of Nos1 suppresses cell proliferation and accelerates differentiation [79]. In addition to its role in development, Nos1 has been previously associated with some aspects of maternal care. Inhibiting Nos1 activity, either using knockout mice or injections of nitric oxide synthase inhibitors, disrupts offspring protection [80,81]. Combined, these results indicate that a significant number of genes associated with neural development are differentially regulated in lactating females compared to virgins, and this compliments previous research in our lab suggesting that the postpartum period may coincide with a terminal differentiation state in specific brain regions [5]. Overall, 64 developmental genes were identified with significantly altered expression in postpartum females (Figure 3). Subsequent studies examining changes in cellular differentiation or structural changes within the postpartum brain would provide more direct evidence for the role of developmental processes in the postpartum brain.

### Enrichment for Ion channel activity in the MPOA

Functional annotation clustering revealed enrichment for genes relating to ion channel activity in lactating compared to virgin MPOA using NIH DAVID. The majority of genes within the cluster, approximately 65%, were down-regulated. Among those identified were 6 potassium channel genes as well as 3 GABA_A_ receptor subunits. Two of the GABA_A_ receptor subunits, Gabre and Gabrq, were down-regulated in microarray results, and this was verified using qPCR for Gabre (p = 0.008), but only a trend toward down-regulation was found for the Gabrq subunit (p = 0.066). GABA_A_ receptors containing Gabre have been shown to exhibit spontaneous and GABA modulated channel activity [82]. Interestingly, a microarray conducted in the LS found a significant up-regulation of both Gabre and Gabrq in postpartum compared to virgin females [5]. These results suggest that GABA_A_ receptor subunit expression is altered in lactating females, though the direction of regulation is region specific. Previous studies have associated GABA signaling in the LS with maternal defense [83], while the MPOA is primarily associated with pup retrieval and nest building behaviors [3]. Collectively, these results indicate that a wide array of genes associated with ion channel activity are altered in the postpartum female, and may contribute to altered neural excitation during lactation.

### Additional changes to genes related to mental health disorders in the postpartum MPOA

Angiotensin converting enzyme (Ace) was significantly down-regulated in lactating females compared to virgin females (p = 0.027), and to our knowledge has not been previously associated with the transition from a virgin to a postpartum state. The brain and pituitary renin-angiotensin system is involved in reproductive behaviors, fluid homeostasis, and oxytocin and vasopressin release from the pituitary [84]. Ace converts angiotensin I to an active form, angiotensin II (AngII), which binds to angiotensin receptors found primarily within brain regions associated with the hypothalamic pituitary adrenal (HPA) axis [85]. In addition, enhanced AngII activity is associated with increased anxiety and depression [86]. Future studies investigating the role of Ace and the brain/pituitary renin-angiotensin system during lactation are necessary to grasp the role this system plays during the postpartum period.

Microarray data indicated that Glul was significantly up-regulated in the postpartum MPOA compared to virgin females, and this was confirmed using qPCR (p = 0.006). This finding is consistent with a previous microarray which showed significant up-regulation of Glul in postpartum females in the septal region [4]. Astrocytic Glul, otherwise known as glutamine synthetase, metabolizes glutamate to glutamine, which can then be utilized for GABA synthesis [87,88]. The role of GABA in MPOA on maternal care is likely to be complex and has been addressed in previous studies [8,89].

### MiRNAs linked to the formation of the postpartum brain

The number of miRNAs linked to the significant microarray results (p < 0.01) was small, numbering only 9 miRNAs with 7 conserved binding sites. When a larger subset of genes was used (p < 0.05), 108 individual miRNAs with 78 conserved binding sites were identified (Additional file 3). A previous microarray study utilizing the septal region of virgin and lactating females identified several miRNA binding sites linked with up-regulation of gene expression in postpartum females. A large number of miRNAs identified in both studies were identical: when p < 0.01, 6 of the 9 miRNAs found in the MPOA were identical to septal miRNAs, and 51 of 108 miRNAs found in the MPOA were identical to septal miRNAs when p < 0.05. However, these comparisons should be interpreted with caution. While this study utilized ToppCluster for detecting enrichment for miRNA binding sites, the septum microarray used Gene Set Enrichment Analysis (GSEA). In addition to different parameters and algorithms utilized by each database, ToppCluster gathers miRNA binding site information from a variety of sources, including the Molecular Signatures Database, TargetScan, and MicroRNA.org. GSEA exclusively uses the Molecular Signatures Database. Despite these limitation, the overlap in miRNAs identified in both studies indicates that some miRNAs may participate in the formation of the postpartum brain. Future studies investigating the expression levels of miRNAs during the virgin and postpartum period would contribute to the current understanding of the construction of the maternal brain.

### Alterations in transcription factor expression and Co-regulation of transcription factors with other genes of interest

A number of transcription factors were differentially regulated between virgin and lactating females (Table 2). Using the Animal Transcription Factor Database [90] we identified 37 transcription factors, 7 chromatin remodeling factors, and 17 co-factors that were differentially regulated between virgin and postpartum females. In an effort to understand direct or indirect links of these transcription factors to large scale gene expression changes, we utilized WGCNA to establish a weighted network of genes that may be co-regulated during the postpartum period. Our results consisted of two individual modules with a large number of co-regulated genes, including the majority of transcription factors, co-factors, and chromatin remodeling factors found in the top 734 genes from the microarray. Further, a number of genes linked to mental health disorders and CNS development already identified during enrichment analysis were correlated to the identified transcription factors, indicating that these genes may be co-regulated in the postpartum MPOA (Additional file 4).

**Table 2:** List of transcription factors, co-factors, and chromatin remodeling factors in significant (p < 0.01) microarray results

Transcription factors						
Arid5b	Cic	Creb1	Dbp	Deaf1	Egr1	Foxr2
Gatad2b	Hmgxb4	Hmg20a	Myc	Nfya	Nr1d1	Nr4a3
Olig2	Pias3	Rfx4	Rfx5	Rora	Smad9	Smarcc2
Sox5	Stat3	Tcfe3	Tef	Tulp4	Zfhx4	Zfp39
Zfp551	Zfp592	Zfp687	Zfp868	Zim1	Zmiz1	Zxdb
Zxdc	5730507C01Rik					
Transcription co-factors						
Atf7ip	Cited1	Cited2	Dcc	Hcfc1	Htt	Ifi202b
Maml3	Optn	Psmc5	Prkcb	Rlim	Sra1	Supt3h
Taf10	Tdp2	Wdr77				
Chromatin remodeling factors						
Chd5	Kdm4d	Mtf2	Pcgf2	Setd1a	Smarca4	

We specifically investigated the module closely correlated with the postpartum phenotype (R^2^ = 0.85, student p-value = 0.0005). The module contained 8 transcription factors, including some previously linked to maternal behaviors and mental health disorders. Knockout mice for two Creb1 isoforms inhibit pup retrieval and nest building behaviors [91], and has been identified as a possible candidate gene for depression [92,93]. In addition, SNPs have been identified in Prkcb that are associated with postpartum depression [94]. Combined, these WGCNA results suggest correlations between differentially expressed transcription factors and other significant genes of interest from our microarray results, which can be analyzed in future studies for their involvement in maternal care at the level of the MPOA.

## Conclusions

This study found altered mRNA expression of specific genes linked to mental health disorders during the transition from a virgin to postpartum state. We were able to show that gene expression changes in the MPOA of lactating versus virgin females are enriched for genes relating to ASD, BPD, depression, and schizophrenia, and confirmed a subset of microarray results using qPCR. This research suggests that genes linked to mental health disorders associated with social deficit may be promoting sociability in the lactating female, and that the maternal brain could provide new insights into understanding social disorders. In addition, enrichment for genes related to CNS development and ion channel activity were found in microarray results. These results contribute to previous evidence that the postpartum brain may be an endpoint for neural development, though future studies examining differentiation and neural maturation in brain regions associated with maternal care are necessary. Combined, these results further our understanding of alterations in gene expression in the postpartum brain, and contribute to our knowledge of this critically important brain region.

## Methods

### Animals

Sixteen nulliparous outbred hsd: ICR (Harlan, Madison, WI) females were approximately 70 days old at the time of the study. One half of the nulliparous females were kept with female littermates, and the remaining half were housed with breeder males (also outbred hsd: ICR) for two weeks. At the end of two weeks, all females used were individually housed for the remainder of the study, except for maternal exposure to pups for 7 days following parturition. This housing strategy provided all females with a similar social environment [95,96]. This paradigm has been used in previous studies to examine gene expression changes that result from a culmination of experiences that ultimately lead to functioning maternal behavior in the postpartum female [4,97]. Outbred mice were utilized so that results would be more broadly applicable to other mouse strains and potentially other rodent models. Mice had ad libitum access to breeder chow (Harlan, Madison, WI) and water, and were kept in polypropylene cages with nestlets that were changed weekly prior to parturition. On postpartum day 0, litters were culled to 11 pups and postpartum females with less than 9 pups were removed from the study. No behavioral tests were conducted on the postpartum or virgin females in this study (n = 6 postpartum and 6 virgin females for microarray analysis). Behavioral tests were avoided because we were interested in natural changes in gene expression between a virgin and postpartum female, and it is possible that behavioral tests could lead to alterations in gene expression. However, future tests examining the correlation between behavior and alterations in gene expression would be valuable in evaluating critically important genes. All females were kept in a 12:12 light/dark cycle with lights on at 6:00 CST. All procedures followed the guidelines of the National Institutes of Health Guide for the Care and Use of Laboratory Animals and were approved by University of Wisconsin Animal Care and Use Committee.

### Tissue collection and RNA extraction

**Figure 5:**
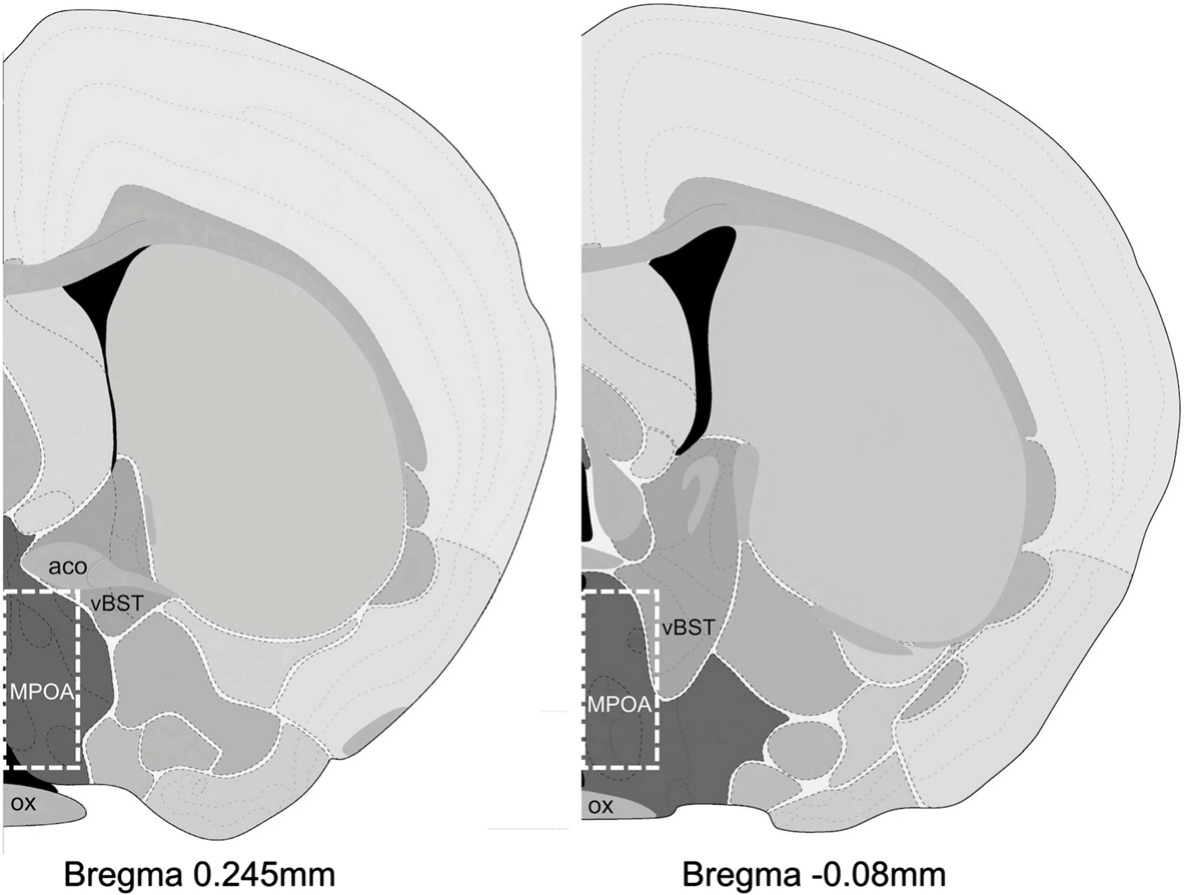
**Representation of the region collected (white boxed area) for microarray and qPCR analysis.** MPOA was collected from approximately Bregma 0.245 mm to -0.08 mm. Images were modified from The Allen Mouse Brain Atlas (coronal sections, version 1, 2008). aco: anterior commissure; MPOA: medial preoptic area; ox: optic tract; vBST: ventral bed nucleus of the stria terminalis.

On postpartum day 7, virgin and postpartum females were lightly anesthetized with isoflurane and decapitated between 9:00 and 12:00 CST. The estrous states of virgin females were determined with a vaginal lavage, and only females in diestrous were used for this study (n = 7). Brains were flash frozen in isopentane and stored at -80 C. Brains were sliced on a cryostat (Leica CM1850, Bannockburn, IL, USA) to a 200 micrometer thickness, mounted on gelatin coated glass slides, and the MPOA was punched out using a micropunch technique and the Brain Punch Set (Stoelting, Wood Dale, IL, USA) under a dissection microscope (Figure 5). Samples from 8 postpartum females and 7 virgin females were collected and frozen at -80 C until RNA extraction. Total RNA was extracted using the Aurum Total RNA Fatty and Fibrous Tissue kit (Bio-Rad, Hercules, CA) with minor alterations to the manufacturers protocol. Briefly, two low stringency washes were added just before RNA elution, and total RNA for all regions was eluted with 30 μL nuclease free water heated to 70°C instead of the elution solution provided by the manufacturer. RNA concentration was determined using a NanoDrop 2000 spectrophotometer (Thermo Scientific, Wilmington, DE, USA), and stored at -80°C until further processing.

### High density oligonucleotide array hybridization

Six samples from each group were randomly selected for the microarray experiment. The total RNA extracted from the MPOA was used with the GeneChip Mouse Gene 1.0 ST array (Affymetrix, Santa Clara, CA). cDNA for array hybridization was synthesized from 200 ng of total RNA using the Ambion GeneChip WT Expression Kit (Ambion, Austin, TX) according to the manufacturer’s specifications. Briefly, double stranded cDNA was synthesized from the total RNA, and then used as a template for the production of single-stranded cRNA synthesis. The cRNA was used as a template for a second round of cDNA synthesis, and the resulting DNA-RNA hybrids were degraded. The cDNA was then fragmented and biotinylated using the Affymetrix WT Terminal Labeling kit (Affymetrix). Labeled cDNA samples were hybridized with the arrays for 16 hours at 45°C, then washed and stained according to the manufacturer’s instructions. Arrays were scanned at 570 nm on an Affymetrix GC3000 G7 Scanner and data was extracted and processed using the Affymetrix Command Console v. 3.1.1.1229. cDNA synthesis, fragmentation, labeling, array hybridization, staining, and scanning were performed by the Gene Expression Center at the University of Wisconsin-Madison.

### Probeset level summarization and microarray statistical analysis

Probeset level summarization and normalization were performed using the PLIER algorithm in Affymetrix Expression Console build 1.2.1.20. The BioConductor package limma v3.14.4 was used to perform an array-specific empirical Bayesian implementation of ANOVA to provide inferential statistics for differential expression between the virgin and postpartum MPOA samples. The nominal and false discovery rate (FDR) p-values were calculated, but due to the low number of genes falling below an FDR p < 0.25, the nominal p-value was used for all statistical analysis. Fold change was calculated for each gene by dividing the ratio of the limma-calculated average postpartum expression coefficient and the average virgin expression coefficient. Any gene with a fold change higher than 1 indicates that expression is increased in maternal females relative to virgin females, while a fold change less than 1 indicates that expression is decreased in lactating females.

### Gene enrichment analysis using the modular single-Set enrichment test (MSET)

Enrichment for genes relating to certain psychological disorders in the most significant microarray results was assessed using MSET. Briefly, gene lists of interest relating to ASD, BPD, depression, and schizophrenia were collected from different gene databases: the Autism Database AutDB [28], the Copenhagen DISEASES database [29], the Genetic Association Database (GAD) [30,31] the HuGE Navigator [32] and the Human Malady Compendium (MalaCards) [33], or gathered from genome wide association studies: Autism GWAS [34], and Autism Novel Genes [35]. An overview of the MSET procedure can be found in Figure 1A, and described in greater detail elsewhere [18]. Briefly, the degree of overlap between disorder gene lists and the top 734 most significant genes from the MPOA microarray (p-value < 0.01) was compared to the number of matches generated from 10,000 randomization tests using the entire MPOA microarray background. Results were considered enriched for genes relating to a certain disorder if there was a significantly higher number of disorder related genes in the microarray results compared to the average results from 10,000 randomizations.

### MicroRNA and transcription factor enrichment analysis

Enrichment for specific gene clusters, as well as microRNA and transcription factor binding sites was determined using NIH DAVID and ToppCluster [23,24]. The top 734 significant genes were input into both sites for analysis. High classification stringency settings were used for NIH DAVID analysis and all ToppCluster results had Bonferroni p-values < 0.05.

Relevant clusters were then input into GeneMania to visualize the connectivity within each individual network. ToppCluster analysis found no enrichment for transcription factor binding sites, but a relatively large number of transcription factors were found to be differentially regulated in the top 734 significant genes. As a result, the Animal Transcription Factor Database was utilized to identify the transcription factors, co-factors, and chromatin remodeling factors that were differentially regulated in the significant microarray results.

### Weighted network construction using WGCNA

The free statistical software R was used for all WGCNA computations [25,26,98]. The top 2389 probes (nominal p < 0.05) were used to calculate a sufficient beta value (beta = 21) to satisfy scale free topology (R^2 > 0.8) for the current analysis. However, since many genes are represented by multiple probes, we have only reported the number of unique genes found within each module. Using unsupervised hierarchical clustering, a minimum module size of 30 genes, and a threshold for merging modules of 0.25, we identified two independent modules (blue and turquoise) from our microarray results. Correlations of module eigengenes to either the postpartum or the virgin state were calculated, as were student p-values for each correlation. We then pruned the original WGCNA output into files containing gene-to-gene correlations with transcription factors for each module. Each module then contained a total of 441 and 136 genes for the turquoise and blue modules, respectively. The blue module, which was correlated with the postpartum phenotype, was then visualized by importing the data into Cytoscape v3.0.1.

### CDNA synthesis and quantitative real-time PCR (qPCR)

To confirm microarray findings, qPCR was performed on 13 genes of interest using MPOA samples from the 8 lactating and 7 virgin females collected. The target genes selected have been researched for their involvement in psychological disorders, and were found on many of the ASD, BPD, depression, and schizophrenia gene lists used for the enrichment analysis. Two stable reference genes, hydroxymethylbilane synthase (Hmbs) and hypoxanthine guanine phosphoribosyl transferase (Hprt), were used for target gene normalization (non-normalized p-values > 0.50).

cDNA was synthesized from 100 ng of total RNA using a SuperScript III First Strand Synthesis System for RT-PCR kit (Invitrogen, Carlsbad, CA) and an Eppendorf MasterCycler Personal PCR Machine (Eppendorf, Hamburg, Germany). Samples were amplified in a Bio-Rad CFX96 Touch Real Time System (Bio-Rad) in triplicate using SsoFast EvaGreen Supermix (Bio-Rad) and primers specific to the target gene (Additional file 5). The thermal profile used is as follows: an initial melting step of 95°C for 30 seconds, followed by 40 cycles of a 95°C melting step for 5 seconds, a 56-58°C annealing step for 20 seconds (see Additional file 5 for specific annealing temperatures for each gene), and a 72°C elongation step for 20 seconds. The relative expression of target genes between the virgin and postpartum females was measured using the relative expression software tool (REST 2009).

### Re-evaluation of microarray normalization and MSET analysis following the finding of unverified qPCR results

A subset of genes from significant microarray results were selected for qPCR verification, but confirmation of significant changes in expression were not found for all genes. Six of the genes tested, Cntnap2, Gabrd, Gabrq, Reln, Shank3, and Tsc1 did not have significant qPCR results, though all trended toward an increase or decrease in expression that was identical to microarray results (data not shown). Previous studies have assessed the correlation between microarray results and qPCR verification, and found a decrease in correlation between microarray data and qPCR results when samples were amplified at later cycles (Ct < 31), which is indicative of a lower copy number gene [99]. Non-significant qPCR results for Gabrd, Reln, and Shank3 may be due to low copy number in the MPOA, since both did not cross the set threshold until after cycle 30. For this study, PLIER was used for microarray normalization, and has been successfully used in three previous microarray studies from our lab [4,5,100]. Our genes of interest were also significant after using RMA normalization, indicating that the normalization technique did not significantly affect microarray results. Microarrays may contain false positives and negatives, which may explain the inconsistent microarray and qPCR data for Cntnap2 and Tsc1. Collectively, these results indicate that confirmation of microarray results is necessary for a thorough analysis of genes of interest.

Four of the six genes that had non-significant qPCR results were found in lists of mental health disorders or enrichment analysis clusters. In order to ensure that enrichment was still significant without these genes, they were removed from the microarray results and all tests were run again. Enrichment for genes relating to CNS development and ion channel activity were still found using ToppCluster and NIH DAVID, respectively. Significant enrichment for mental health disorders were detected in all lists tested except for a BPD list, which was identical to our initial findings (data not shown). This indicates that our enrichment results in the MPOA are robust and can withstand the removal of some genes of interest, furthering the observation that genes linked to mental health disorders and CNS development are found in high numbers in the MPOA of postpartum versus virgin females.

## Competing interests

The authors declare that they have no competing interests.

## Authors’ contributions

Conceived of the experiments: SCG and TMD. Performed the experiments: CZ, SAS and TMD. Analyzed the data: BEE, MCS, and TMD. Contributed reagents, materials, and other tools used in the study: SCG. Wrote the paper: TMD and SCG. All authors read and approved the final manuscript.

## Supplementary Material

Additional file 1 Complete list of all MPOA microarray target genes, nominal p-values, and fold changes. Click here for file

Additional file 2 All genes relating to ASD, BPD, depression, and schizophrenia identified in significant microarray results. Click here for file

Additional file 3 **List of all miRNAs significantly linked to microarray genes with a p < 0.05.** In the list, if a dash separates two letters, it indicates all miRNAs within those letters (e.g. MIR-30A-E indicates MIR-30A, MIR-30B, MIR-30C, MIR-30D, and MIR-30E). Click here for file

Additional file 4 **List of the top WGCNA generated correlations between significant transcription factors (nominal p < 0.01) and other significant genes (nominal p < 0.05) from the MPOA microarray.** The blue module, which was correlated with the postpartum phenotype, is listed first and followed by the turquoise module. Click here for file

Additional file 5 Primer sequences and annealing temperatures for all genes analyzed using real-time qPCR. Click here for file
