# Supplementary material for: Genes showing altered expression in the medial preoptic area in the highly social maternal phenotype are related to autism and other disorders with social deficits
Source: BMC Neurosci. 2014 Jan 14;15:11. doi: 10.1186/1471-2202-15-11 (PMC3906749; doi:10.1186/1471-2202-15-11)
Supplement: Additional file 2 — All genes relating to ASD, BPD, depression, and schizophrenia identified in significant microarray results. [file 1471-2202-15-11-S2.doc]

| ASD Related Genes | | | | | | |
| --- | --- | --- | --- | --- | --- | --- |
| Acox3 | Acsl4 | Adam23 | Alox5ap | Amigo2 | Ankrd11 | Apbb2 |
| Ass1 | Atp1b2 | Atp2b2 | Atp10a | Baiap3 | Bcl2 | Bcam |
| Cacna1g | Cdh13 | Cdyl2 | Chrna3 | Churc1 | Cldn1 | Clstn2 |
| Clstn3 | Cntnap2 | Cpeb1 | Creb1 | Csn1s1 | Dab1 | Dab2ip |
| Dnajc11 | Dock3 | Dok5 | Dscam | Elmo1 | Erc2 | Exoc2 |
| Flt1 | Gabra2 | Gbp4 | Gna11 | Gpr56 | Grik1 | Grik3 |
| Hcrtr2 | Htt | Igf1 | Igfbp3 | Irs2 | Kcnj10 | Lphn2 |
| Mfge8 | Mfsd6 | Myc | Ncan | Nos1 | Nova1 | Nrp2 |
| Nxph1 | Oprl1 | Oxtr | Park2 | Pcdh9 | Pcdh11x | Plxdc2 |
| Podxl2 | Ppapdc1a | Prkcb | Ptchd1 | Ptprm | Rai14 | Rap1gap2 |
| Rfx4 | Rgs16 | Rims3 | Rora | Rps6ka6 | Runx1t1 | Ryr2 |
| Setd1a | Setd7 | Shank3 | Slc23a2 | Slc30a1 | Slc41a3 | Sntg2 |
| Socs2 | Sorbs1 | Spon1 | Susd4 | Syt17 | Tnip2 | Trim9 |
| Trpc3 | Tsc1 | Ush1c | Vgf | Vldlr | Wbscr17 | Wnk3-ps |
| BBP Related Genes | | | | | | |
| 1810010H24Rik | Ace | Ache | Alg9 | Bai3 | Bcl2 | Chrna3 |
| Chrnb2 | Cntnap2 | Creb1 | Crim1 | Cry1 | Cry2 | Dbp |
| Dcc | Dock3 | Dnmt1 | Dok5 | Dscam | Dusp6 | Fbn1 |
| Fbxw7 | Fkbp5 | Gab2 | Gabra2 | Gabre | Gabrq | Grik3 |
| Igf1 | Itih3 | Lrrc8c | Ncan | Nefl | Nos1 | Notch3 |
| Nova1 | Nr1d1 | Nr4a3 | Oxtr | Penk | Per3 | Podx12 |
| Rfx4 | Rora | Ryr2 | Slc1a4 | Smyd3 | Trpc3 | Trpc5 |
| Zdhhc8 | Zmiz1 |  |  |  |  |  |
| Depression Related Genes | | | | | | |
| Ace | Acsl4 | Adcyap1r1 | Alkbh3 | Alox5ap | Ampd2 | Bcl2 |
| Bcl9 | Cdh13 | Chrna3 | Creb1 | Cry1 | Cry2 | Dbp |
| Deaf1 | Fkbp5 | Gabra2 | Gabre | Gabrq | Grik1 | Grik3 |
| Hdac5 | Hspa1a | Htra1 | Htt | Itih3 | Kdr | Nos1 |
| Nr1d1 | Nup214 | Nxph1 | Oxtr | Pam | Per3 | Ppm1a |
| Rora | Smpd1 | Sntg2 | Stat3 | Tnfrsf11b |  |  |
| Schizophrenia Related Genes | | | | | | |
| Ace | Ache | Acsl4 | Alg9 | Ankrd11 | Atp2b2 | Bcl9 |
| Cdc25a | Cdc42se2 | Cdh13 | Chi3l1 | Chrna3 | Chrnb2 | Clstn2 |
| Cnih2 | Cntnap2 | Creb1 | Cry2 | Csn1s1 | Dbp | Dcc |
| Dnmt1 | Egr1 | Fbxl21 | Fkbp5 | Fxyd6 | Gabra2 | Gbp4 |
| Glul | Gpr88 | Grik1 | Grik3 | Hmgcs1 | Hspa1a | Igf1 |
| Igfbp3 | Itih3 | Kcnj10 | Lrrc7 | Mfge8 | Ncan | Nos1 |
| Notch3 | Nova1 | Nr1d1 | Nr4a3 | Nt5c2 | Olig2 | Oxtr |
| Padi2 | Pcdh9 | Pcdh11x | Penk | Per3 | Pla2g4c | Ppp1r9b |
| Rgs10 | Rgs16 | Rims3 | Shank3 | Slc1a4 | Slc6a9 | Slc6a11 |
| Slc7a10 | Smpd1 | Ston2 | Tacr3 | Tspan18 | Ucp2 | Vgf |
| Vldlr | Zdhhc8 | Zdhhc15 |  |  |  |  |
