# Supplementary material for: Genes showing altered expression in the medial preoptic area in the highly social maternal phenotype are related to autism and other disorders with social deficits
Source: BMC Neurosci. 2014 Jan 14;15:11. doi: 10.1186/1471-2202-15-11 (PMC3906749; doi:10.1186/1471-2202-15-11)
Supplement: Additional file 3 — List of all miRNAs significantly linked to microarray genes with a p < 0.05. In the list, if a dash separates two letters, it indicates all miRNAs within those letters (e.g. MIR-30A-E indicates MIR-30A, MIR-30B, MIR-30C, MIR-30D, and MIR-30E). [file 1471-2202-15-11-S3.doc]

| MIR-9 | MIR-15A-B | MIR-16 | MIR-17 | MIR-19A-B |
| --- | --- | --- | --- | --- |
| MIR-20A-B | MIR-27A-B | MIR-30A-E | MIR-33A-B | MIR-34A |
| MIR-34C-5P | MIR-92 | MIR-93 | MIR-96 | MIR-101 |
| MIR-106A-B | MIR-124 | MIR-128-A-B | MIR-129-5P | MIR-130A-B |
| MIR-140-3P | MIR-141 | MIR-144 | MIR-145 | MIR-181A-D |
| MIR-182 | MIR-195 | MIR-200A-C | MIR-203 | MIR-204 |
| MIR-211 | MIR-218 | MIR-299-3P | MIR-301A-B | MIR-338-5P |
| MIR-339 | MIR-340 | MIR-369-3P | MIR-374-A-B | MIR-377 |
| MIR-410 | MIR-421 | MIR-424 | MIR-429 | MIR-448 |
| MIR-449A-B | MIR-452 | MIR-454 | MIR-466 | MIR-485-3P |
| MIR-497 | MIR-501-3P | MIR-506 | MIR-513A-3P | MIR-519A-D |
| MIR-519-3P | MIR-519C-3P | MIR-520D | MIR-522 | MIR-548A-5P |
| MIR-548B-5P | MIR-548C-5P | MIR-548D-5P | MIR-548E,G-J,P | MIR-559 |
| MIR-561 | MIR-570 | MIR-586 | MIR-646 | MIR-655 |
| MIR-656 | MIR-768-3P | MIR-1200 | MIR-1264 | MIR-1271 |
| MIR-1274A | MIR-1283 | MIR-1305 |  |  |
