# Supplementary material for: Genes showing altered expression in the medial preoptic area in the highly social maternal phenotype are related to autism and other disorders with social deficits
Source: BMC Neurosci. 2014 Jan 14;15:11. doi: 10.1186/1471-2202-15-11 (PMC3906749; doi:10.1186/1471-2202-15-11)
Supplement: Additional file 5 — Primer sequences and annealing temperatures for all genes analyzed using real-time qPCR. [file 1471-2202-15-11-S5.doc]

| Gene Symbol | Gene Name | NCBI Accession Number | Annealing Temp |  | Primer Sequence |
| --- | --- | --- | --- | --- | --- |
| Ace | Angiotensin I converting enzyme | NM_009598.2 | 57.5ºC | Forward | 5’-GACAACCTGGAGCAAGACC-3’ |
|  |  |  |  | Reverse | 5’-AGGGAACGACGCACATAG-3’ |
| Bcl2 | B cell leukemia/lymphoma 2 | NM_009741.3 | 57.5ºC | Forward | 5’-GAGTGGGATGCTGGAGATG-3’ |
|  |  |  |  | Reverse | 5’-GCTGGAAGGAGAAGATGCC-3’ |
| Cntnap2 | Contactin associated protein-like 2 | NM_001004357.2 | 57.5ºC | Forward | 5’-AGCCTCCGACATTCTCTTTC -3’ |
|  |  |  |  | Reverse | 5’-TTGCCCCTTTTCTCATTTCC-3’ |
| Gabrd | Gamma-aminobutyric acid (GABA) A receptor, subunit delta | NM_008072.2 | 57.5ºC | Forward | 5’-CTGCTACGTCTTTGTGTTTGC-3’ |
|  |  |  |  | Reverse | 5’-CCGTTTCTTCCTGTAGTCGG-3’ |
| Gabre | Gamma-aminobutyric acid (GABA) A receptor, subunit epsilon | NM_017369.2 | 57ºC | Forward | 5’-GACCTGGTATGATGAGCGTC-3’ |
|  |  |  |  | Reverse | 5’-CATCCTTGTGGATGAGAGCC-3’ |
| Gabrq | Gamma-aminobutyric acid (GABA) A receptor, subunit theta | NM_020488.1 | 57.5ºC | Forward | 5’-GGAGAGTTGTTGCCCGATAC-3’ |
|  |  |  |  | Reverse | 5’-TCCTGCACATTTGCTACCAC-3’ |
| Glul | Glutamate-ammonia ligase | NM_008131.3 | 58ºC | Forward | 5’-TGAGAGAACCATCCTATTCACTG-3’ |
|  |  |  |  | Reverse | 5’-TAAGCAGTAATGAAGCTGAGACC-3’ |
| Hmbs | Hydroxymethylbilane synthase | NM_001110251.1 | 56.5ºC | Forward | 5’-CCCTTGTGATGCTGTTGTC-3’ |
|  |  |  |  | Reverse | 5’-GCGGGTGTTGAGGTTTC-3’ |
| Hprt | Hypoxanthine guanine phosphoribosyl transferase | NM_013556.2 | 57ºC | Forward | 5’-GGCTTACCTCACTGCTTTC-3’ |
|  |  |  |  | Reverse | 5’-TTGCTGGAGTTGCTGAAGAG-3’ |
| Nos1 | Nitric oxide synthase I, neuronal | NM_008712.2 | 57ºC | Forward | 5’-GCCACCAATGAGAAAGAGAAG-3’ |
|  |  |  |  | Reverse | 5’-TAGTAGCGAGGTTGTAGCAG-3’ |
| Oxtr | Oxytocin receptor | NM_001081147.1 | 58ºC | Forward | 5’- CTGACTTGGGGTAGGGAAATG-3’ |
|  |  |  |  | Reverse | 5’- AGGGTAGTAGAAATAGGTGGGAG-3 |
| Reln | Reelin | NM_011261.2 | 58.5ºC | Forward | 5’-GACTGGGATGTGGTAAAGGTG-3’ |
|  |  |  |  | Reverse | 5’-TTGCTGGAGTTGCTGAAGAG-3’ |
| Shank3 | SH3/ ankyrin domain gene 3 | NM_021423.3 | 57.5ºC | Forward | 5’-TGGAGTAGAGGAGGCTGAC-3’ |
|  |  |  |  | Reverse | 5’-CTGTGGAAATGGTGCTTGTG-3’ |
| Socs2 | Suppressor of cytokine signaling 2 | NM_001168655.1 | 57ºC | Forward | 5’-TGCTGGACCAAACACAAACG-3’ |
|  |  |  |  | Reverse | 5’-GCCTTCTTTCACCTTCCCAAA-3’ |
| Tsc1 | Tuberous sclerosis I | NM_022887.3 | 58.5ºC | Forward | 5’-CACTGTTGGCTCACTTCCC-3’ |
|  |  |  |  | Reverse | 5’-GTCACGCTGTCCTCATCAC-3’ |
